# Benchmarking Visual Detection Tiers for Event-Centric Edge Intelligence in Maritime Disaster Response

**DOI:** 10.3390/s26185764

**Published:** 2026-09-10

**Authors:** Sobirjon Habibullaev, Juno Choi

**Affiliations:** GreenBlue Corporate Research Institute, GreenBlue Inc., 40 Imi-ro, Uiwang-si 16006, Gyeonggi-do, Republic of Korea

**Keywords:** maritime edge intelligence, object detection, YOLO26, YOLO11, YOLOv8, Jetson AGX Orin, Raspberry Pi 5, reComputer AI R2000-12, computer vision benchmark

## Abstract

Rapid maritime incident response requires visual recognition near the point of observation, but practical deployment must balance detection accuracy, inference latency, hardware-conversion constraints, and limited communication capacity. This study benchmarks the visual-compute component of an Event-Centric Edge Intelligence (ECEI) architecture on two maritime edge tiers: a Jetson AGX Orin 64 GB Developer Kit and a reComputer AI R2000-12 based on Raspberry Pi 5. Three tasks were selected to represent distinct maritime event categories: Human Detection for person-overboard response, six-class Ship Detection for vessel monitoring and collision awareness, and eleven-class Marine Trash Detection for floating-debris and pollution monitoring. YOLO26, YOLO11, and YOLOv8 were evaluated across 54 model–dataset–device configurations. Repeated on-device validation used 100 deterministically selected images per task and 20 measured repetitions for each checkpoint, yielding 108,000 timed predictions. On the paired S/N subset, the median reComputer/AGX mean-latency ratio was 4.40×; mean latency ranged from 19.84–57.09 ms on AGX and 68.89–172.11 ms on reComputer. The highest AGX $mAP_{50–95}$ values were 0.331, 0.813, and 0.639 for Human, Ship, and Marine Trash Detection, respectively; the highest reComputer values were 0.318, 0.806, and 0.558. Across the evaluated event-message configuration, event-candidate JSON bytes were 98.20% lower than raw JPEG bytes. These results support deployment-aware visual-tier selection and an auditable detector-to-event interface for maritime edge intelligence.

## 1. Introduction

Maritime accidents and environmental incidents—including person-overboard events, collisions, drifting debris, and floating pollution—require fast recognition under constrained communications and heterogeneous sensing. Public maritime incident datasets further support the operational relevance of these categories: Korea Coast Guard maritime distress records include vessel accidents, person-related distress events, casualties, rescued persons, deaths, missing persons, accident causes, weather, and location fields [1], while Korea Coast Guard marine pollution accident records include occurrence time, location, coordinates, pollution source, pollutant type, and spill volume [2]. A cloud-only pipeline can provide centralized analytics, but it also introduces a dependency on uplink availability and moves time-critical perception away from the operational site. Edge computing addresses this architectural tension by placing computation near data sources, thereby enabling local decisions and selective upstream reporting [3,4].

The operational case for edge intelligence is especially strong in the maritime domain. Optical cameras provide rich semantic information, while Automatic Identification System (AIS) messages and radar can supply complementary identity and kinematic context [5,6,7,8,9,10]. The present study isolates visual detection so that calibration, temporal alignment, tracking, association, event fusion, and communication can be examined under dedicated protocols. Detector *mAP* therefore supports visual-compute selection, while bandwidth, robustness, operational latency, and response quality are designated as system-level evaluation targets.

This study preserves the ECEI research direction for distributed maritime disaster response while concentrating the empirical analysis on visual object detection across two edge-device classes. In this article, “benchmarking” refers to a controlled deployment-aware evaluation protocol rather than an isolated comparison of detector accuracy. The evaluation target is not a detector model alone, but a model–dataset–device deployment combination. The protocol fixes the maritime task definitions, YOLO model families and scales, device tiers, training controls, validation-image selection, repeated inference measurement, and reporting metrics so that visual-compute operating points can be compared reproducibly across heterogeneous maritime edge platforms. Accordingly, the novelty of this work is not a new detector architecture, but the integration of accuracy, latency distribution, hardware constraints, and detector-to-event payload evidence into a tier-selection framework for maritime edge intelligence.

Three operationally important maritime tasks were selected: Human Detection for person-overboard response, six-class Ship Detection for vessel monitoring and collision awareness, and eleven-class Marine Trash Detection for floating-debris and pollution monitoring. YOLO26, YOLO11, and YOLOv8 were evaluated at L, M, S, and N scales on the Jetson AGX Orin and at S and N scales on the reComputer. This tier-specific coverage directly represents the intended deployment roles of the two platforms.

The contributions of this article are:

1. A deployment-aware visual-tier benchmark that separates the measured optical detector path from future downstream event-fusion and communication functions;
2. A factorial training benchmark comprising 54 model–dataset–device configurations, complemented by 108,000 repeated complete-call on-device inference measurements from deterministically hash-ranked images;
3. A paired analysis of detection accuracy, wall-clock training time, convergence, and inference-latency distributions summarized by mean, P50, P95, standard deviation, and throughput; and
4. An accuracy–latency Pareto operating analysis, a documented Hailo-8 deployment constraint with a PyTorch CPU baseline, and auditable detector-output/event-candidate JSON records for interface-level payload measurement.

## 2. Related Work

### 2.1. Edge Computing and Deployment-Aware Evaluation

Edge computing places computation near sensing to reduce response delay and dependence on wide-area connectivity [3,4]. Surveys of edge AI further frame model placement as a joint systems-and-learning problem rather than a simple migration of cloud workloads [11]. Recent device studies reinforce that model scale alone is insufficient: runtime, quantization, power mode, postprocessing, and end-to-end pipeline overhead can change the accuracy–efficiency ranking on Hailo-8 and Jetson platforms [12,13]. Reproducible edge-SoC benchmarks further show that deployment performance depends on factors beyond nominal model size or compute rating, including device-specific execution paths, memory behavior, framework overhead, and practical deployment constraints [14].

### 2.2. Maritime Detection Datasets and Benchmarks

Early maritime benchmarks exposed the importance of explicit splits, consistent class mappings, and domain-specific evaluation [15]. MaCVi 2025 expanded public maritime protocols to distance estimation and embedded obstacle segmentation, and MaCVi 2026 added thermal, multimodal, and vision-to-chart tracks [16,17]. These benchmarks show that maritime perception must address tiny or ambiguous objects, reflections, clutter, weather, and target-scale variation while documenting deployability constraints.

### 2.3. Embedded YOLO and Accelerator Validation

Single-stage YOLO detectors are attractive for embedded perception because model scales expose useful accuracy–computation trade-offs. Recent USV-oriented work improves lightweight YOLOv8 for multiscale marine targets [18], while MSJA-Net combines multiscale feature enhancement, attention, and segmentation-assisted augmentation for ships [19]. The present benchmark compares YOLOv8, YOLO11, and YOLO26 as implemented in Ultralytics [20,21,22], emphasizing measured $mAP_{50–95}$ and runtime instead of assuming that similarly named losses or nominal FLOPs are comparable across software generations.

Target-specific accelerator validation connects model conversion, calibration, quantization, compiled execution, retained accuracy, and runtime measurement. Published Hailo studies document INT8 calibration, Hailo Executable Format generation, post-quantization accuracy, latency, and power [12,23]. The present reComputer measurements establish only the PyTorch CPU reference point. Under the available study environment, custom YOLO checkpoints could not be compiled into .hef artifacts because the required Hailo Dataflow Compiler workflow depends on a supported Linux x86_64 build host that was not available. Accordingly, Hailo-8 is treated as a deployment constraint and future acceleration pathway rather than as an empirically measured runtime in this article.

### 2.4. AIS–Vision Fusion and the ECEI Boundary

Recent video–AIS systems combine specialized visual detection with calibration, tracking, temporal alignment, and identity association for vessel geolocation [8]. Other work uses AIS motion priors to recover visually lost or deformed tracks during occlusion [9], while multimodal remote-sensing pipelines cross-reference optical or SAR detections with AIS to investigate non-reporting vessels [10].

These studies motivate the downstream ECEI concept and clarify its interface with the present work. The contribution here is a visual-tier operating benchmark that supplies measured detector outputs and compute-tier choices to a subsequent end-to-end fusion study.

## 3. Event-Centric Edge Intelligence Architecture

**Figure 1:**
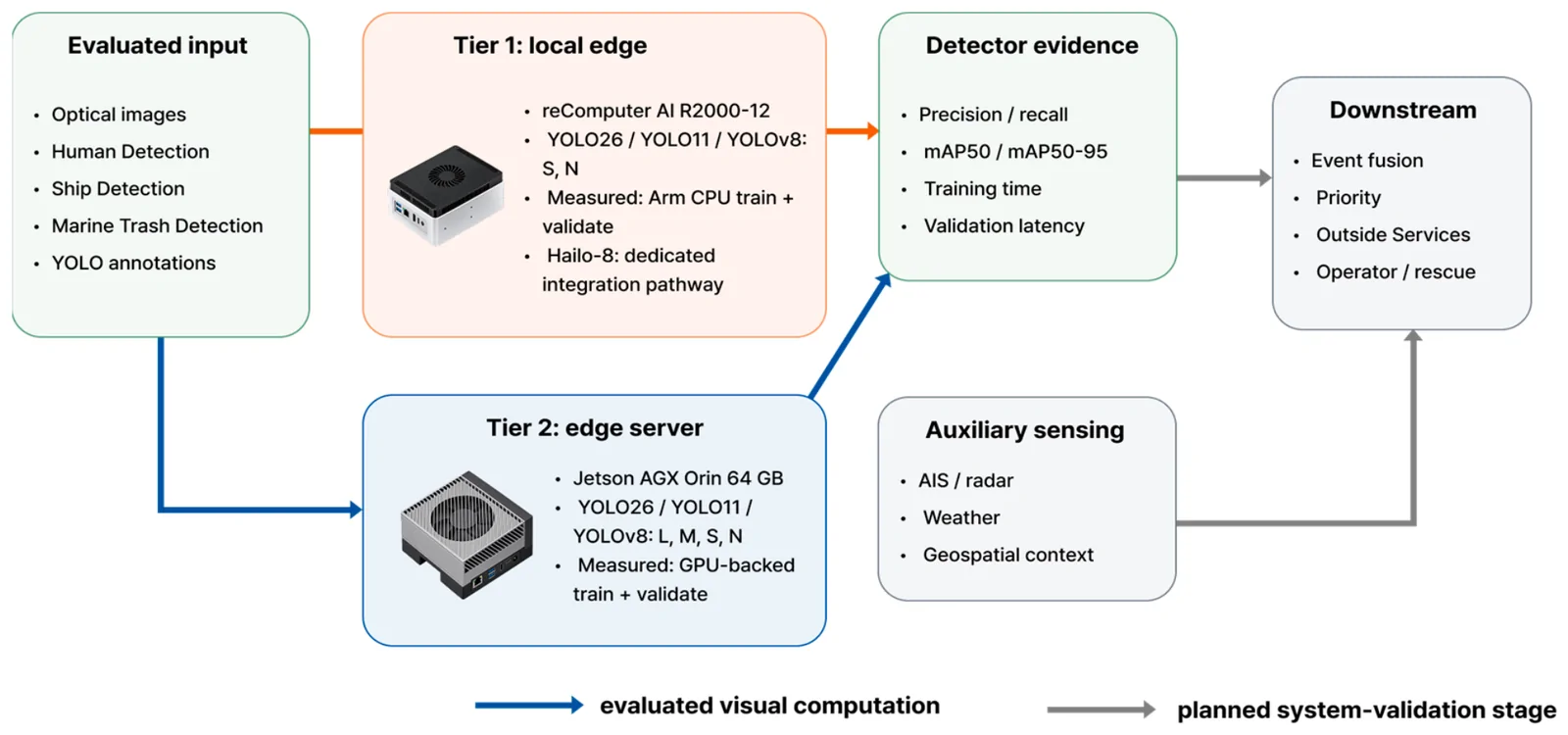
ECEI visual-compute benchmark and system boundary. The blue path denotes the evaluated visual-computation path on the higher-capacity Tier 2 edge platform, while the orange path denotes the evaluated lightweight visual-screening path on the Tier 1 local edge node. Dashed gray paths denote the planned system-validation stages for auxiliary sensing, event fusion, priority assignment, and operational response.

Figure 1 positions the detector benchmark within the broader ECEI system. Tier 1 is a deployable local edge node represented by the reComputer AI R2000-12. Tier 2 is a higher-capacity coastal or vessel-side edge server represented by the Jetson AGX Orin. Optical detections can be converted into event objects and later combined with auxiliary vessel, environmental, and geospatial observations. A minimal event record contains an event identifier, timestamp, location, object class or track, confidence, evidence reference, priority, and provenance.

### 3.1. Tiered Computation

The architecture separates high-throughput visual processing from lightweight local screening. The AGX tier hosts all four selected model scales and is suited to continuous or multi-stream processing; the reComputer tier covers S/N models and is suited to lower-rate, duty-cycled, or event-triggered screening. Together, these tier-specific choices constitute the accuracy–latency operating policy.

### 3.2. Mathematical Formulation of the ECEI Pipeline

Equations (1)–(7) provide an author-defined formalization of the proposed ECEI pipeline and define the system boundary for this study. They are not taken directly from a single prior study; rather, they organize established concepts from temporal sensor alignment, reliability-aware fusion, visual detection, event representation, and edge communication into a common framework. Their verification status and the empirically measured scope of this article are specified in Section 3.3.

Let S={vis,vessel,env,distress} denote the available sensing modalities, where vessel represents optional positioning and track observations. For modality k, observations arrive at timestamps $T_{k}$ with an estimated latency $\delta_{k}$. At reference time t, the most temporally compatible observation is selected by

(1) $$\tau_{k}^{*}\left(t\right)=\arg\underset{\tau \in T_{k}}{\min}\left|\tau -\left(t-\delta_{k}\right)\right|,\;\left|\tau_{k}^{*}\left(t\right)-\left(t-\delta_{k}\right)\right|\leq \epsilon_{k}$$

where $\epsilon_{k}$ is the admissible age tolerance. Each accepted observation is encoded by a modality-specific function $f_{k}$. Its contribution is conditioned on a reliability descriptor $r_{t}^{k}$ that may represent timestamp freshness, signal quality, visibility, or sensor status:

(2) $$h_{t}^{k}=f_{k}\left(x_{t}^{k}\right),\;\alpha_{t}^{k}\frac{\exp\left(\psi_{\theta }\left(h_{t}^{k},r_{t}^{k}\right)\right)}{\sum_{j\in S_{t}}\exp\left(\psi_{\theta }\left(h_{t}^{j},r_{t}^{j}\right)\right)}$$

The normalized weights satisfy $\sum_{k\in S_{t}}\alpha_{t}^{k}=1$. The fused representation and event-class probabilities are then

(3) $$h_{t}=\sum_{k\in S_{t}}\alpha_{t}^{k}h_{t}^{k},\;p_{t}=g_{\theta }\left(h_{t}\right)$$

For the visual branch evaluated in this work, a detector produces $D_{t}=\{\left(y_{i},b_{i},c_{i}\right){\}}_{i=1}^{N_{t}}$, where $y_{i}$ is the predicted class, $b_{i}$ is the bounding box, and $c_{i}$ is confidence. In the complete architecture, detections and contextual observations are converted into semantic event candidates by

(4) $$E_{t}=\{e_{i}=\left(y_{i},c_{i},l_{i},t,m_{i}\right):c_{i}\geq \gamma \}$$

where $l_{i}$ is the estimated event location, $m_{i}$ is provenance and supporting metadata, and γ is the event-admission threshold. For operational ordering, the proposed priority score is a convex combination of confidence, urgency, spatial risk, and contextual severity:

(5) $$\rho_{i}=w^{⊤}{\left[c_{i},u_{i},s_{i},v_{i}\right]}^{T},\;w\geq 0,\;1^{T}w=1$$

Here, $c_{i}$ denotes the detector confidence of event candidate i, while $u_{i}$, $s_{i}$, and $v_{i}$ denote normalized urgency, spatial risk, and contextual severity, respectively. The constraint w≥0 ensures that each component contributes non-negatively, and $1^{T}w=1$ makes the score a convex combination. The operational weights are reserved for outcome-calibrated system evaluation.

If $Z_{t}^{m}$ denotes the semantic event set emitted by edge node m, the node-level event set is defined as the union of node-level event sets:

(6) $$Z_{C}\left(t\right)={⋃}_{m=1}^{M}Z_{t}^{m}$$

Two system-level quantities follow directly from this representation: the fractional communication reduction η_D_ and the latency $L\left(t\right)$:

(7) $$\eta_{D}\left(t\right)=1-\frac{B_{e}\left(t\right)}{B_{r}\left(t\right)},\;L\left(t\right)=T_{C}\left(t\right)-T_{\mathrm{obs}}\left(t\right)$$

$B_{e}\left(t\right)$ and $B_{r}\left(t\right)$ are the event-message and raw-stream byte volumes, respectively; $T_{\mathrm{obs}}\left(t\right)$ is the observation time and $T_{C}\left(t\right)$ is the time at which the event becomes available to the central platform.

To avoid overclaiming system-level validation, Table 1 summarizes the verification status of Equations (1)–(7) by separating conceptual ECEI definitions, empirically measured detector/interface quantities, and prospective end-to-end system metrics. Quantitative conclusions are restricted to the measured quantities; the remaining expressions structure subsequent system validation.

### 3.3. Scope of Validation

The present validation separates conceptual ECEI system definitions from empirically measured quantities. Equations (1)–(3), (5) and (6) define prospective functions for temporal observation selection, reliability-weighted encoding, multimodal fusion, priority scoring, and multi-node event aggregation; these components are reserved for future synchronized multimodal and operational validation. In contrast, the empirical validation in this article centers on the visual detector path and the detector-to-event interface. The measured quantities include detector precision, recall, $mAP_{50}$, $mAP_{50–95}$, completed and selected epochs, wall-clock training time, and repeated complete-call inference latency. Each checkpoint is characterized by mean, median (P50), 95th-percentile latency (P95), standard deviation, and throughput over 2000 timed predictions. Equation (4) is evaluated at the interface level through detector-to-event JSON replay, and Equation (7) is evaluated only for message-level event/raw byte reduction. Camera-to-alert latency, transport performance, serialization time, event-level F1, false-alarm rate, operator response time, and rescue outcomes remain outside this study.

## 4. Materials and Methods

### 4.1. Research Questions and Study Design

The benchmark addresses five research questions:

RQ1. How do YOLO26, YOLO11, and YOLOv8 model family and scale affect detection quality across the three maritime tasks?

RQ2. For the common S/N subset, how do the two edge platforms differ in wall-clock training time and repeated on-device inference-latency distributions?

RQ3. Which configurations occupy useful accuracy–latency operating points for a tiered ECEI deployment?

RQ4. What deployment constraint prevents custom YOLO checkpoints from being evaluated through HailoRT on the reComputer platform under the available study environment?

RQ5. How should detector outputs be encoded as auditable ECEI event-candidate records, and how should message-level payload reduction be measured without claiming end-to-end bandwidth or serialization-time reduction?

The training experiments were conducted to empirically validate the visual detection stage of ECEI and to determine how detector accuracy, training cost, and validation latency depend on both edge platform and model scale. Evaluating 54 model–dataset–device configurations provides evidence for selecting credible models for each tier and for assigning continuous or multi-stream processing to the AGX tier and lightweight or event-triggered screening to the reComputer tier. The experiment therefore validates the visual-compute component rather than the complete end-to-end ECEI pipeline.

The training design is a descriptive factorial benchmark: AGX contains 3 datasets × 3 model families × 4 sizes = 36 configurations, and reComputer contains 3 datasets × 3 families × 2 sizes = 18 configurations. The repeated inference protocol was designed to estimate runtime variability for archived checkpoints, not training-seed uncertainty. Therefore, random seed was not used as the replication unit for inference. Instead, deterministic SHA-256 ranking selected a fixed task-specific image subset so that both platforms were evaluated on identical frames. Every checkpoint was then measured on 100 images × 20 repetitions after 20 separate warm-up iterations. Runtime variability is therefore summarized empirically by mean, P50, P95, standard deviation, and throughput rather than by a single framework observation. Training-seed generalization would require multiple independently trained checkpoints for each configuration under different random seeds. Because the present study uses one archived checkpoint per configuration, training accuracy should be interpreted as configuration-level benchmark evidence rather than statistical generalization over training seeds.

**Figure 2:**
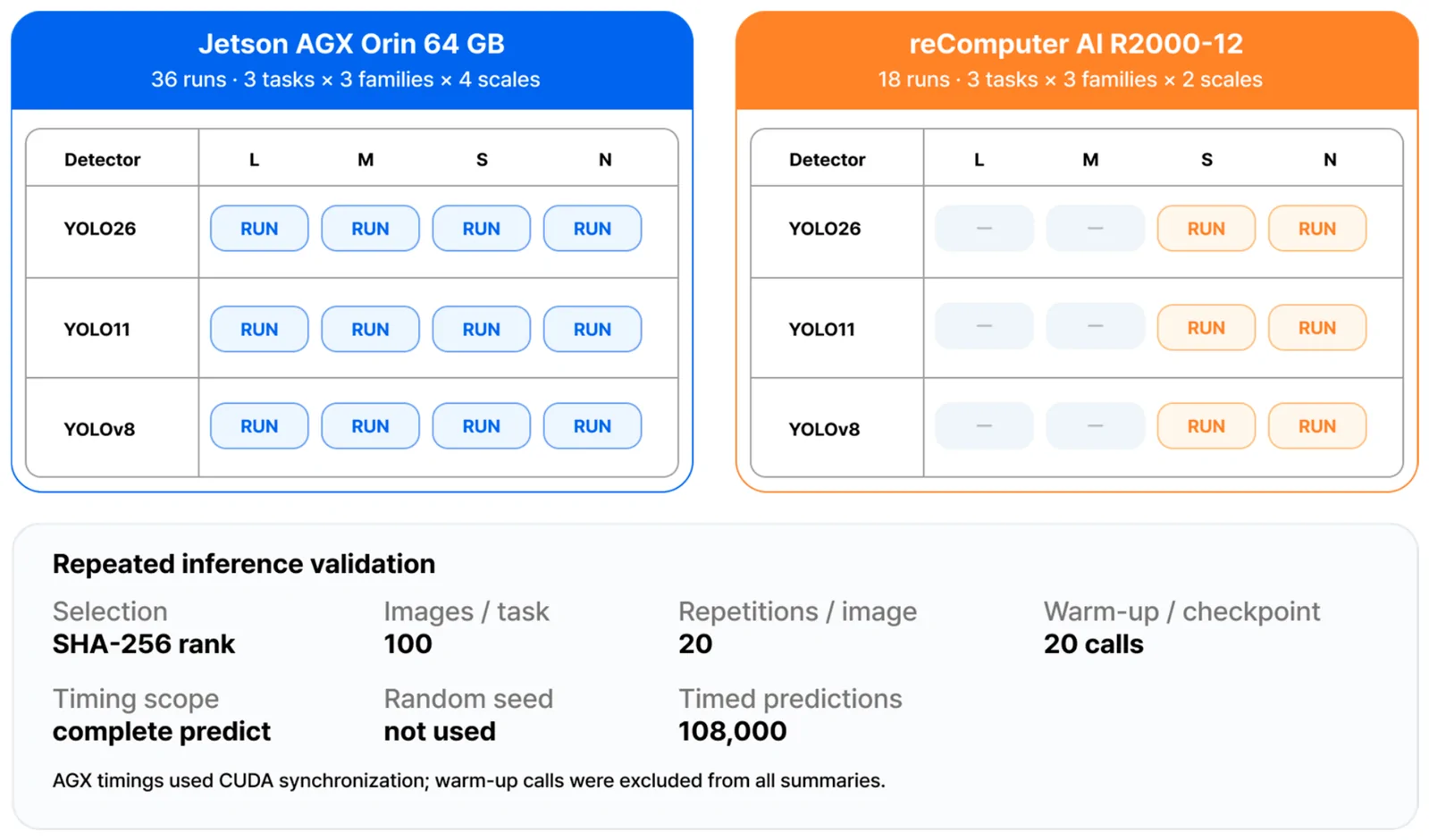
Controlled model coverage and repeated-validation design. AGX covered L/M/S/N and reComputer covered S/N. Repeated inference used deterministic SHA-256 image ranking, 20 warm-up calls per checkpoint, and 20 measured repetitions per image; a random seed was not used for inference selection.

Figure 2 summarizes the controlled model coverage and repeated-validation design used in the benchmark.

### 4.2. Hardware Selection and Compute-Tier Coverage

The two platforms were chosen to instantiate the intended compute tiers of ECEI. The Tier 2 platform was a Jetson AGX Orin 64 GB Developer Kit (NVIDIA Corporation, Santa Clara, CA, USA), representing a high-capacity coastal or vessel-side edge server able to host the complete L/M/S/N model set and support continuous, multi-stream processing. NVIDIA documents this configuration with a 12-core Arm CPU, an Ampere GPU with 2048 CUDA cores, and up to 275 TOPS of nominal AI performance [24]. The Tier 1 platform was the reComputer AI R2000-12 (Seeed Studio, Shenzhen, China), representing a deployable local edge node for lightweight or event-triggered screening with S/N models. It is an enclosed Raspberry Pi 5-class computer with 16 GB memory and a Hailo-8 inference accelerator rated at 26 TOPS [25]. The nominal ratings provide hardware context; the reComputer measurements in the benchmark define the PyTorch CPU reference point, and accelerator characterization follows the staged protocol in Section 4.7. The hardware platforms and exact measured execution paths are detailed in Table 2.

This platform pair provides complementary deployment coverage: the AGX platform represents the higher-capacity tier across the complete model-scale matrix, while the enclosed reComputer represents the compact local tier and provides a practical integration point for future Hailo-8 acceleration once a compatible compilation path becomes available.

### 4.3. Datasets

The benchmark uses three consistently named tasks: Human Detection, Ship Detection, and Marine Trash Detection. The three tasks were intentionally selected to represent different maritime event categories and detection difficulties. Human Detection captures person-overboard or rescue-related visual sensing, Ship Detection captures vessel monitoring and collision-awareness support, and Marine Trash Detection captures floating-debris and pollution monitoring. This task selection is consistent with public maritime incident records: Korea Coast Guard maritime distress data document vessel accidents, person-related distress events, casualties, rescue outcomes, accident causes, weather, and location fields, while marine pollution accident records document occurrence time, location, coordinates, pollution source, pollutant type, and spill volume [1,2]. The tasks differ in object scale, class count, annotation density, object geometry, and background clutter, allowing the benchmark to test whether model-family and device-tier choices remain stable across operationally different maritime scenarios. Human Detection and Ship Detection use version 1 datasets from Roboflow Universe [26,27]. Marine Trash Detection uses the Enhanced Marine Debris Image Data, version 1.1, distributed through AI Hub [28]. Table 3 reports the fixed dataset partitions used in the benchmark.

Human Detection uses a single person-in-water class represented by normalized YOLO bounding boxes. The Ship Detection source provides YOLO polygon annotations; polygon-enclosing boxes were used for the detection benchmark. Its six categories are bulk cargo carrier, container ship, fishing boat, general cargo ship, ore carrier, and passenger ship. Marine Trash Detection uses normalized YOLO bounding boxes for glass, metal, net, PET bottle, plastic buoy, Chinese plastic buoy, miscellaneous plastic, rope, Styrofoam box, Styrofoam buoy, and Styrofoam piece. The originating AI Hub resource also provides bounding-box and segmentation annotations [28].

The validation partitions contain 11,888 annotations for Human Detection in 522 images, 1438 annotations for Ship Detection in 1389 images, and 2211 annotations for Marine Trash Detection in 280 images. The Roboflow sources [26,27] are distributed under CC BY 4.0 and should be obtained from their original versioned pages. The AI Hub source [28] follows the provider’s access and usage terms, and redistribution of the raw data or project-specific eleven-class derivative requires separate provider authorization.

**Figure 3:**
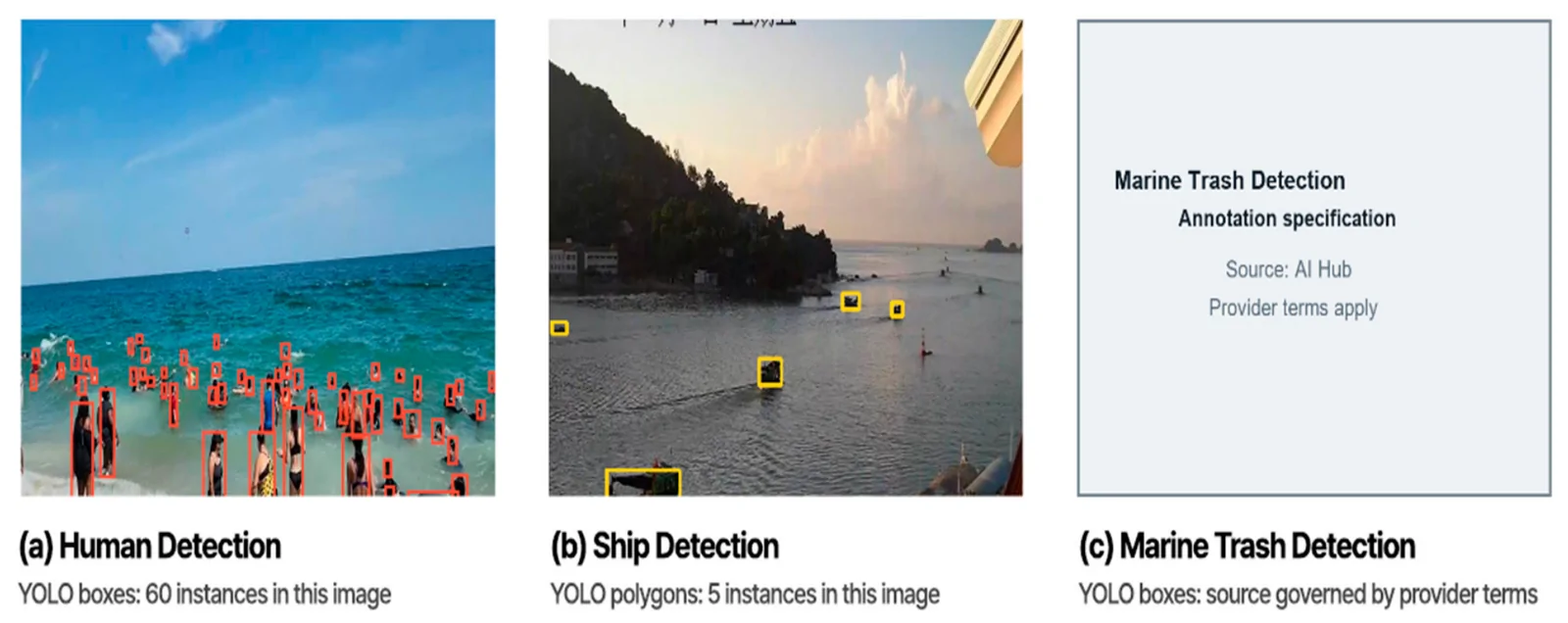
Representative annotation characteristics. Panels (**a**,**b**) illustrate Human Detection boxes and Ship Detection polygons from the cited CC BY 4.0 sources [26,27]. Panel (**c**) is not a source-image example; it summarizes the Marine Trash Detection annotation specification because the source imagery is available through AI Hub [28] under the provider’s access and usage terms.

Figure 3 summarizes representative annotation characteristics of the three benchmark datasets, including bounding boxes, polygon annotations, and the Marine Trash Detection annotation specification.

### 4.4. Model Families and Training Protocol

The selected families were YOLO26, YOLO11, and YOLOv8, with L/M/S/N scales on AGX and S/N scales on reComputer. The archived checkpoint-training runs used an epoch cap of 100, batch size 2, patience 5, image size 320 × 320, deterministic execution, zero data-loading workers, automatic mixed precision where supported, and the Ultralytics automatic optimizer policy. The 320 × 320 input size imposes a common low-compute edge-screening condition across both tiers; the results should be interpreted as compact edge-screening performance rather than maximum attainable detector accuracy. The policy selected AdamW with momentum 0.9 and initial learning rates of 0.002 for Human Detection, 0.001 for Ship Detection, and 0.000667 for Marine Trash Detection.

Table 4 summarizes the fixed training controls and repeated inference-validation design used in the benchmark.

### 4.5. Metrics and Selection Rules

Precision *P* and recall *R* are defined from true positives (*TP*), false positives (*FP*), and false negatives (*FN*). Average precision (*AP*) integrates the precision–recall curve for a class; *mAP* averages AP across classes. $mAP_{50}$ uses an intersection-over-union threshold of 0.50, whereas $mAP_{50–95}$ averages thresholds from 0.50 to 0.95 in 0.05 increments, consistent with common object-detection evaluation [29,30].

(8) $$P=TP/\left(TP+FP\right),R=TP/\left(TP+FN\right)$$

(9) $$IoU\left(B_{p},B_{r}\right)=\frac{\left|B_{p}\cap B_{r}\right|}{\left|B_{p}\cup B_{r}\right|}$$

For class *c* and IoU threshold τ, $AP_{c}\left(\tau \right)$ is the area under the interpolated precision–recall curve. With *C* classes and *T* = {0.50, 0.55, …, 0.95}, the reported aggregate metric is

(10) $$\begin{array}{l} AP_{c}\left(\tau \right)=\int_{0}^{1}P_{c}\left(R;\tau \right)\;dR\\ mAP_{50–95}=\frac{1}{10C}\sum_{\tau \in T}\sum_{c=1}^{C}AP_{c}\left(\tau \right) \end{array}$$

For the archived training runs, the selected checkpoint is the epoch with maximum validation $mAP_{50–95}$, and completed epochs and wall-clock training time are obtained from epoch-level outputs. The latency analysis replaces the earlier single framework-reported timing with a separate repeated on-device protocol applied to the archived best checkpoints.

Inference latency denotes the elapsed time of the complete Ultralytics prediction call on a pre-decoded image, including framework preprocessing, model execution, and postprocessing while excluding file read and image decode. CUDA synchronization was performed immediately before and after each AGX timing. Both devices used time.perf_counter_ns(). For every device–task–checkpoint stratum, 20 warm-up calls preceded 100 images × 20 measured repetitions in round-robin image order. Mean, P50, P95, sample standard deviation, and throughput were computed from the 2000 measured calls. End-to-end camera-to-alert latency remains a separate system metric in Equation (7). Within each task × device stratum, an accuracy–latency Pareto point is a configuration that is not dominated by another configuration in both $mAP_{50–95}$ and repeated mean latency, with at least one strict improvement. Let $q_{i}$ denote a configuration, $m_{i}$ denote the $mAP_{50–95}$ of configuration $q_{i}$, and $l_{i}$ denote its repeated mean latency.

(11) $$q_{j}≻q_{i}⟺m_{j}\geq m_{i},\;l_{j}\leq l_{i},\;\mathrm{and}\;\left(m_{j}>m_{i}\;\mathrm{or}\;l_{j}<l_{i}\right)\;$$

A Pareto point is any configuration $q_{i}$ for which no other configuration $q_{j}$ dominates it within the same task × device stratum.

### 4.6. Reproducibility and Software Environment

To ensure reproducibility and reflect realistic edge deployment conditions, the software environments were tailored to the native OS constraints of each respective hardware platform. The Jetson AGX Orin environment relies on NVIDIA JetPack, which intrinsically bounds the environment to Python 3.8.10 and requires a platform-specific, hardware-accelerated PyTorch build (2.1.0a0+41361538. nv23.06) to leverage its Ampere GPU via CUDA 11.4 and cuDNN 8.6.0.

Conversely, the reComputer AI R2000-12 (Raspberry Pi 5 CM5) operates on a more recent aarch64 Linux distribution natively supporting Python 3.11.2. Consequently, its validation utilized PyTorch 2.11.0+cpu operating strictly in CPU-only mode. Accordingly, the reported reComputer/AGX latency ratios should be interpreted as deployment-stack ratios rather than hardware-only ratios. They combine hardware throughput, backend availability, operating-system constraints, PyTorch builds, and Ultralytics runtime behavior. This interpretation is consistent with the deployment-oriented objective of the benchmark, but it does not isolate the independent contribution of hardware alone.

Table 5 summarizes the software environments used for training and validation on each benchmark platform.

### 4.7. Hailo-8 Deployment Constraint and CPU Baseline Definition

The reComputer AI R2000-12 integrates a Hailo-8 accelerator designed for executing precompiled neural network graphs. However, custom YOLO checkpoints generated by the training pipeline are PyTorch .pt files, whereas HailoRT execution requires quantized and hardware-allocated Hailo Executable Format (.hef) artifacts. Generating .hef artifacts requires the Hailo Dataflow Compiler, which depends on a supported Linux x86_64 build environment.

Under the available study environment, a compatible Linux x86_64 Hailo compilation path was not available. Therefore, the custom YOLO checkpoints could not be converted into .hef artifacts for HailoRT execution. The reComputer measurements in this study consequently define a PyTorch CPU baseline for S/N models on the ARM64/aarch64 platform. They do not characterize Hailo-8 accelerated inference, INT8 quantization retention, HailoRT throughput, or accelerator power efficiency.

Future HailoRT evaluations should include documented compiler and runtime versions, .pt-to-.hef conversion records, output-equivalence checks between PyTorch and HailoRT execution, and power/thermal logging for the compiled artifacts.

### 4.8. Repeated On-Device Validation Design

The repeated validation used the same SHA-256-ranked set of 100 images per task on both devices. Image ranking is deterministic and content-independent; it is not a pseudorandom training seed. Twenty warm-up calls per checkpoint were excluded, followed by 20 measured repetitions per image. This produced 72,000 AGX and 36,000 reComputer observations. Table 6 reports the Pareto candidates recalculated with repeated mean latency.

### 4.9. Event-Candidate Encoding and Payload Measurement Protocol

To connect checkpoint-derived detections to the event-centric ECEI layer without overclaiming end-to-end system performance, the offline replay writes one detector-output envelope and one event-candidate envelope per frame. Each accepted tuple (class, bounding box, confidence) is mapped to an event record containing event_id, event_type, timestamp, source_node, sensor_type, object_class, confidence, bounding_box, location, priority, priority_rule, event_admission_rule, evidence_ref, and provenance. Person, ship, and marine-trash detections map to person-overboard, vessel-presence, and floating-debris candidates, respectively; zero-detection frames retain an auditable empty event_candidates array.

The same SHA-256-ranked subset of 100 original JPEGs per task (300 unique frames) was reused for every applicable checkpoint. All 54 logical configurations were replayed at image size 320, confidence 0.50, NMS IoU 0.70, maximum 300 detections, and batch size 1. Detector and event records were serialized as compact, key-sorted UTF-8 JSON without a BOM, indentation, or trailing newline; raw size was the unchanged JPEG file length. The primary message-level reduction is 100 × (1 − Σ event JSON bytes/Σ raw JPEG bytes). Camera acquisition, transport headers, retransmission, streaming policy, and alert latency remain outside this measurement.

No retraining was performed for the payload experiment; predictions were regenerated from the archived best checkpoints and validation JPEGs. The detector-to-event transformation was evaluated through an offline replay using compact JSON serialization. Therefore, the payload experiment evaluates schema conformance and serialized byte size rather than on-device event-serialization performance. The AGX and reComputer labels identify checkpoint origin rather than separate serialization-speed measurements. SHA-256 audit found 37 unique weight states among 54 logical slots because 17 AGX/reComputer pairs were byte-identical. Retaining all logical slots produced a 98.20% event/raw reduction; a unique-weight sensitivity analysis produced 98.18%.

## 5. Results

### 5.1. Run Completion and Convergence

All 54 planned configurations completed with evaluable validation metrics. Forty-nine runs ended before the 100-epoch cap and five reached 100 epochs. Under patience = 5, an early-stopped run ordinarily completes approximately 5 epochs after its selected optimum. Appendix A therefore uses completed epochs as the universal endpoint and reports selected epochs where emitted by the framework.

Training duration followed dataset size, stopping epoch, model complexity, and device throughput. The longest observed run was YOLOv8-S for Human Detection on reComputer (120.746 h), whereas the shortest was YOLOv8-N for Marine Trash Detection on AGX (0.970 h). Because completed epoch counts differ, the paired analysis treats these durations as operational outcomes under the common early-stopping rule.

### 5.2. Detection Accuracy

Table 7 summarizes the best observed accuracy point for each task and device together with its repeated latency statistics. The table shows that model selection is task-dependent rather than universal: the best detector family differs between Human/Ship Detection and Marine Trash Detection. This supports the deployment-oriented interpretation that model generation or size alone should not determine tier selection.

Performance leadership varied by task. On AGX, YOLOv8-M achieved the highest $mAP_{50–95}$ for Human Detection (0.331) and Ship Detection (0.813), while YOLO26-M led Marine Trash Detection (0.639). On reComputer, YOLOv8-S was highest for all three tasks (0.318, 0.806, and 0.558). This task-dependent leadership indicates that no YOLO family was uniformly superior across maritime sensing tasks. A plausible explanation is that Human Detection is dominated by small, partially occluded, irregular targets under waves, foam, glare, and reflections, while Ship Detection contains more geometrically structured objects and fewer classes. Marine Trash Detection, by contrast, contains eleven fine-grained debris classes with diverse object scales, materials, and background clutter. Under the common 320 × 320 edge-screening condition, these differences can change the effective accuracy–capacity trade-off among model families. This interpretation remains empirical, and a class-, size-, occlusion-, and weather-stratified error analysis is reserved for subsequent validation.

The reComputer maxima for Human Detection and Ship Detection were numerically close to the AGX maxima and are interpreted as device-specific operating points. The model coverage and execution backends differ by tier, so the comparison supports deployment selection rather than cross-platform equivalence. The observed S-scale results demonstrate feasible training on the reComputer with the reported wall-clock requirements.

### 5.3. Training-Time Comparison on the Paired S/N Subset

**Figure 4:**
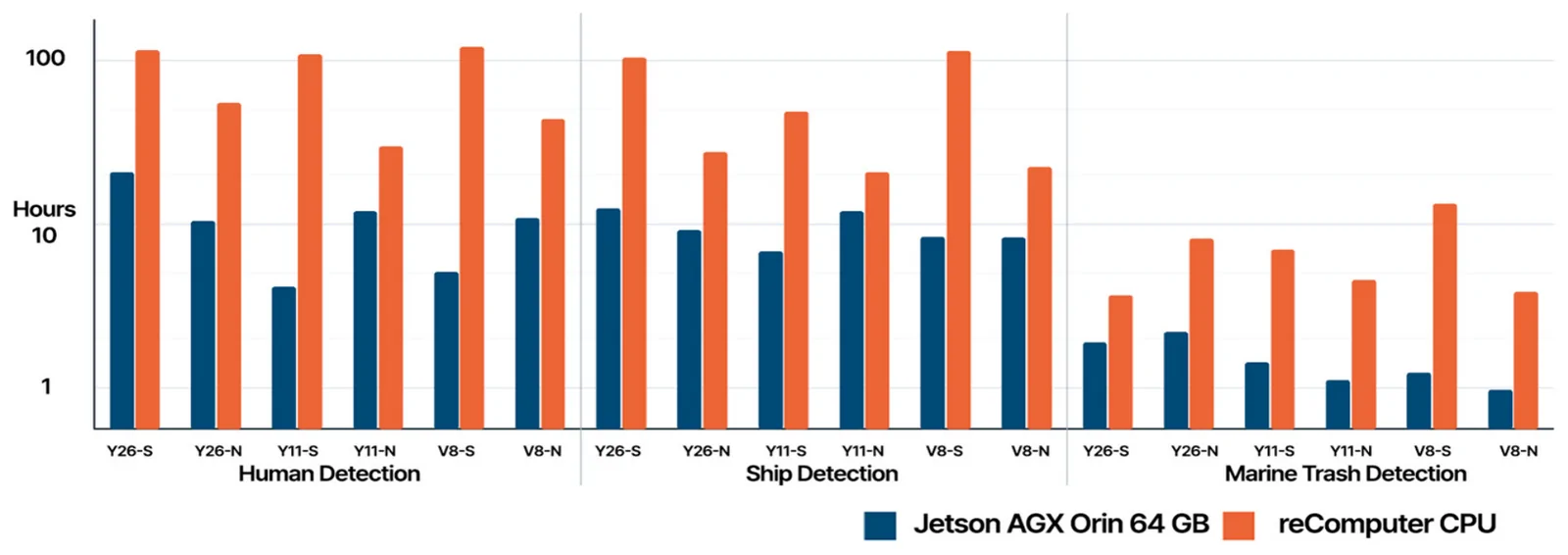
Wall-clock training time for the 18 matched S/N configurations across Human Detection, Ship Detection, and Marine Trash Detection. The vertical axis is logarithmic.

Figure 4 should be read as an operational training-time comparison for the configurations that are common to both devices. The logarithmic axis is used because the observed training times span a wide range across tasks, model scales, stopping epochs, and devices. The figure shows that reComputer training is feasible for S/N models but generally requires substantially longer wall-clock time than AGX training.

Across all 18 pairs, the median reComputer-to-AGX training-time ratio was 4.51×, with a range from 1.73× for the Ship Detection YOLO11-N configuration to 26.22× for the Human Detection YOLO11-S configuration. The variation reflects both device throughput and early-stopping behavior. For example, the Human Detection YOLO11-S configuration completed 28 epochs on AGX and 100 epochs on reComputer, which increased the observed wall-clock ratio. The ratios characterize operational time under the common stopping rule; a per-epoch throughput analysis provides a complementary view.

### 5.4. Repeated On-Device Inference Latency

**Figure 5:**
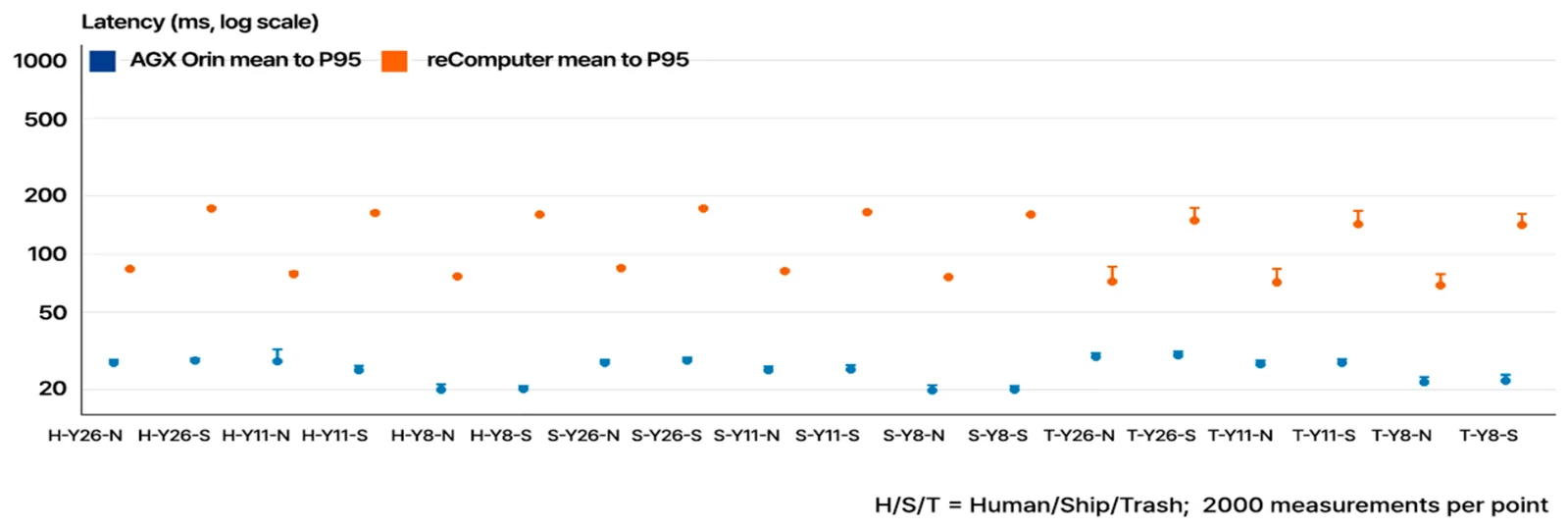
Repeated complete-call inference latency for the 18 matched S/N configurations. Points indicate mean latency and upper whiskers indicate P95 latency over 2000 measured calls per checkpoint. The vertical axis is logarithmic.

Figure 5 shows the latency distribution of the common S/N configurations under repeated on-device inference. The mean points represent typical complete-call inference time, while the P95 whiskers show high-percentile latency that is relevant to operational response stability. The figure shows that AGX consistently provides lower complete-call latency, whereas reComputer CPU remains within a lightweight screening range for S/N models.

Across 108,000 measured predictions, AGX mean latency ranged from 19.84 to 57.09 ms, P95 from 20.82 to 61.46 ms, and throughput from 17.52 to 50.39 FPS. The corresponding reComputer CPU ranges were 68.89–172.11 ms, 76.95–174.84 ms, and 5.81–14.52 FPS. Across the 18 paired S/N configurations, the median reComputer/AGX ratio was 4.40× for mean latency (range 2.45–7.99×) and 4.59× for P95 latency (range 2.51–7.76×). The fastest mean was 19.845 ms for Ship Detection YOLOv8-N on AGX; the fastest reComputer mean was 68.888 ms for Marine Trash Detection YOLOv8-N. Marine Trash Detection showed the widest CPU tails, including a 42.66 ms P50-to-P95 gap for YOLO26-S.

Table 8 aggregates the repeated latency ratios by task and shows that the reComputer/AGX gap is consistent across the paired S/N subset but varies by task. The ratios should be interpreted as deployment-stack ratios rather than hardware-only ratios because each platform uses its native operating system, PyTorch build, and execution backend. This distinction is important for deployment planning: the values indicate expected latency differences under the evaluated software–hardware stacks, not an isolated comparison of processor capability alone.

### 5.5. Accuracy–Latency Operating Points

**Figure 6:**
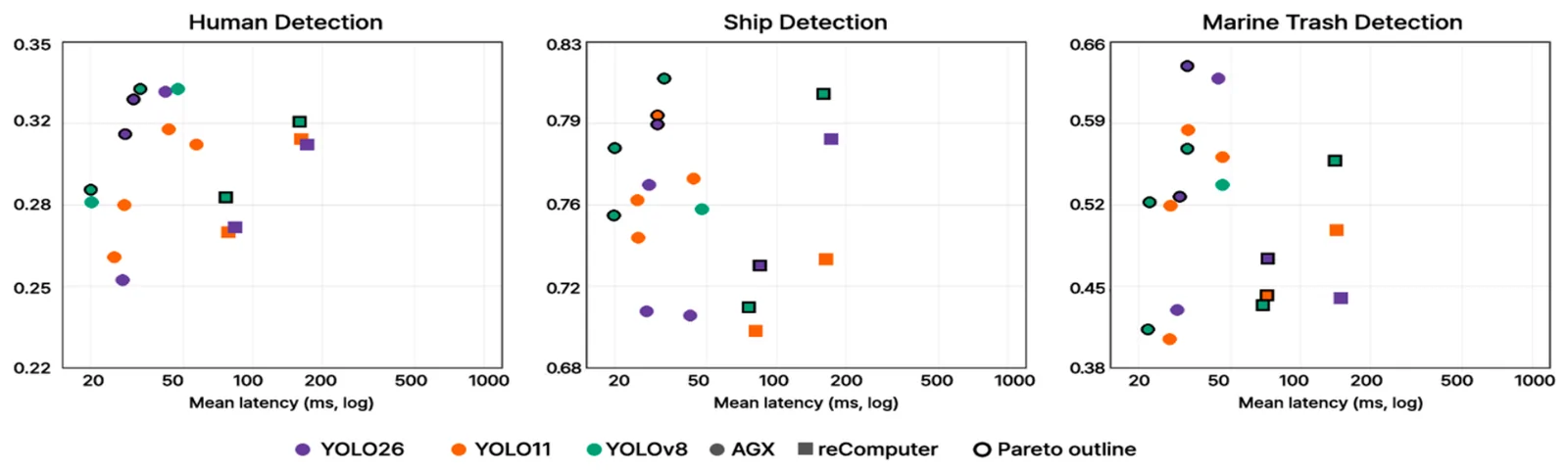
Accuracy–latency trade-off across all 54 checkpoint configurations using repeated mean complete-call latency. Outlined points are non-dominated within each task and device.

Figure 6 should be interpreted as an operating-point map rather than a simple model ranking. The horizontal axis represents repeated mean complete-call latency, and the vertical axis represents $mAP_{50–95}$. Outlined points are non-dominated configurations within each task and device stratum, meaning that no other configuration in the same stratum provides both equal-or-higher accuracy and equal-or-lower latency with at least one strict improvement. The figure shows that the largest model is not always the most useful deployment choice: N-scale models anchor low-latency points, while task-dependent S/M candidates enter the frontier when additional computation is acceptable. Table 6 lists these recalculated operating choices; they should not be interpreted as statistically superior models.

### 5.6. Event-Candidate Payload Interface

The completed offline CPU replay produced and independently verified 5400 detector-output JSON files and 5400 event-candidate JSON files containing 27,663 accepted detections. Event JSON totaled 17,182,188 bytes versus 952,012,854 raw JPEG bytes, yielding 98.20% aggregate reduction; detector JSON alone yielded 99.53%. Task reductions were 89.77% for Human Detection, 98.33% for Ship Detection, and 99.29% for Marine Trash Detection. The checkpoint-origin aggregates were 98.06% (AGX) and 98.46% (reComputer). These byte measurements describe serialized content and exclude serialization time, edge-device runtime, and network transport.

Table 9 shows that event-candidate encoding substantially reduces serialized message size compared with raw JPEG transmission across all tasks. The aggregate event/raw reduction is 98.195%, with larger reductions for Ship Detection and Marine Trash Detection than for Human Detection because the admitted event records and source JPEG sizes differ by task. These values should be interpreted as message-level byte reductions only; they do not include transport headers, retransmission, serialization latency, or camera-to-alert delay.

Table 10 clarifies the model-level payload behavior on the paired S/N subset. For all 18 shared S/N configurations, accepted detections, mean event payload, and event/raw reduction were identical between AGX-origin and reComputer-origin entries because their checkpoint files were byte-identical. Payload size therefore follows admitted detections and serialized record content rather than device execution speed; a smaller payload should not be interpreted by itself as better detector performance.

**Figure 7:**
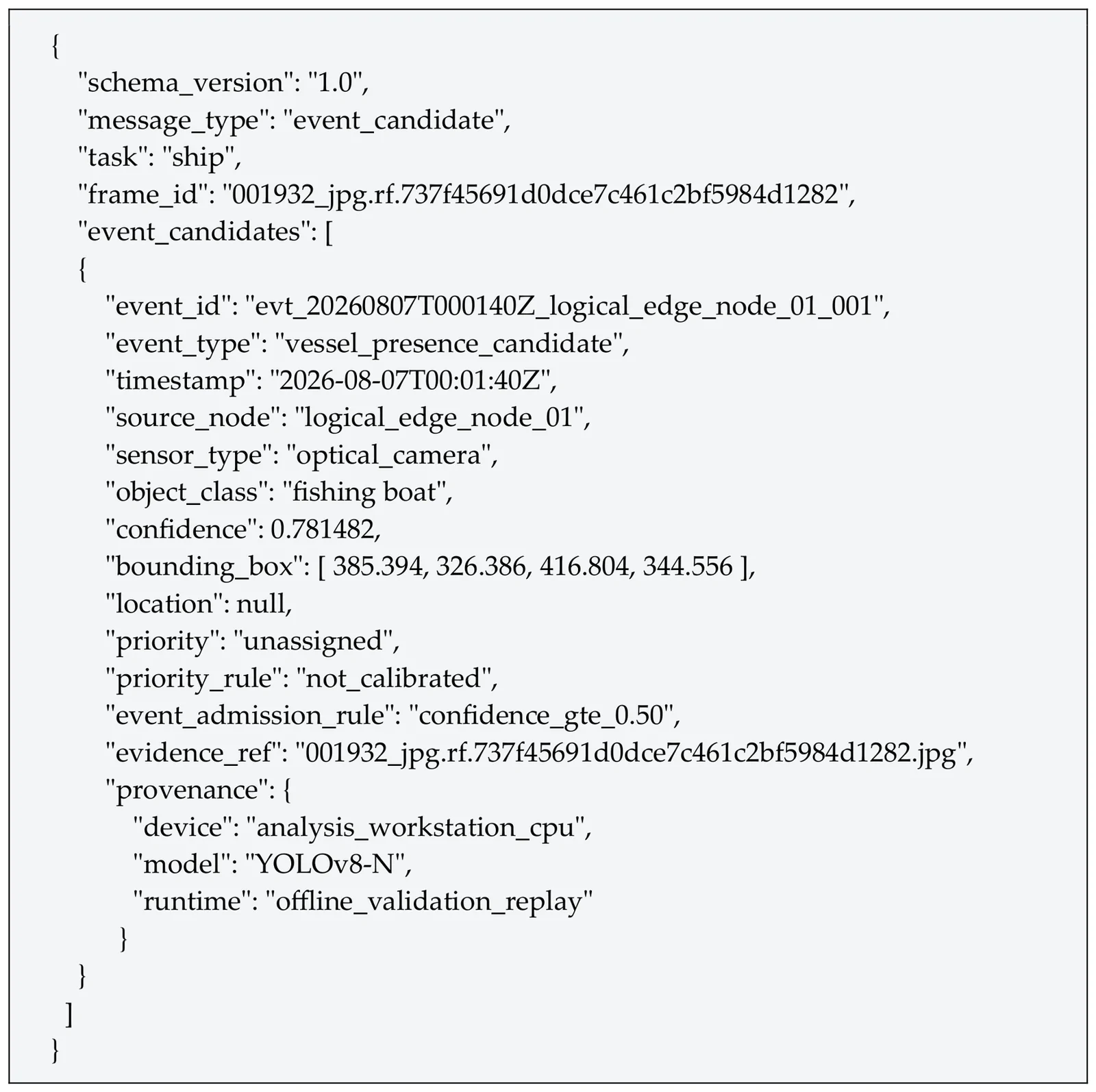
Abbreviated overview of the implemented event-candidate schema. The complete detector-output and event-candidate JSON records are provided in Listings 1 and 2. source_node is logical ECEI metadata, provenance records the actual offline replay context, and timestamps are deterministic serialization placeholders rather than camera acquisition times.

Figure 7 presents an abbreviated overview of the implemented event-candidate schema used in the offline replay. The diagram is intended to clarify the interface fields rather than to imply that all downstream ECEI functions were operationally deployed. To avoid ambiguity, the complete detector-output and event-candidate JSON records are provided in Listings 1 and 2. The source_node field identifies a logical edge node, the provenance field records the offline replay context, and the timestamp field is a deterministic placeholder because calibrated camera acquisition times were not available in the source archive.

### 5.7. Concrete Detector and Event JSON Records

Listing 1 and 2 provide a concrete record-level example of the detector-to-event transformation. Listing 1 shows the original detector-output envelope, including image metadata, class label, confidence score, and pixel-space bounding-box coordinates. Listing 2 shows the corresponding ECEI event-candidate envelope, where the accepted detection is mapped to a vessel_presence_candidate and augmented with event metadata, evidence reference, and replay provenance. These examples are included to make the JSON interface auditable and to clarify which fields are measured, assigned, or left uncalibrated in the present study.

Listing 1 **Complete detector-output JSON example (Ship Detection, YOLOv8-N, one accepted detection).**

{

  “confidence_threshold”: 0.5,

  “detections”: [

    {

      “bounding_box”: [

        385.394,

        326.386,

        416.804,

        344.556

      ],

      “class_id”: 2,

      “class_name”: “fishing boat”,

      “confidence”: 0.781482

    }

  ],

  “frame_id”: “001932_jpg.rf.737f45691d0dce7c461c2bf5984d1282”,

  “image_height”: 640,

  “image_name”: “001932_jpg.rf.737f45691d0dce7c461c2bf5984d1282.jpg”,

  “image_width”: 640,

  “message_type”: “detector_output”,

  “schema_version”: “1.0”,

  “task”: “ship”

}

Listing 2 **Complete ECEI event-candidate JSON generated from the detector record in Listing 1.**

{

  “event_candidates”: [

    {

      “bounding_box”: [

        385.394,

        326.386,

        416.804,

        344.556

      ],

      “confidence”: 0.781482,

      “event_admission_rule”: “confidence_gte_0.50”,

      “event_id”: “evt_20260807T000140Z_logical_edge_node_01_001”,

      “event_type”: “vessel_presence_candidate”,

      “evidence_ref”: “001932_jpg.rf.737f45691d0dce7c461c2bf5984d1282.jpg”,

      “location”: null,

      “object_class”: “fishing boat”,

      “priority”: “unassigned”,

      “priority_rule”: “not_calibrated”,

      “provenance”: {

        “device”: “analysis_workstation_cpu”,

        “model”: “YOLOv8-N”,

        “runtime”: “offline_validation_replay”

      },

      “sensor_type”: “optical_camera”,

      “source_node”: “logical_edge_node_01”,

      “timestamp”: “2026-08-07T00:01:40Z”

    }

  ],

  “frame_id”: “001932_jpg.rf.737f45691d0dce7c461c2bf5984d1282”,

  “message_type”: “event_candidate”,

  “schema_version”: “1.0”,

  “task”: “ship”

}

The timestamp is a deterministic replay placeholder, source_node is logical metadata, location is null, and priority is unassigned because the source archive provides neither acquisition time nor calibrated operational geolocation or priority labels.

## 6. Discussion

### 6.1. Answers to the Research Questions

RQ1—model accuracy. Model family and size interacted with task. YOLOv8-M led Human Detection and Ship Detection on AGX, while YOLO26-M led Marine Trash Detection. YOLOv8-S led all three detection tasks on reComputer. These results reject a universal ranking of detector families and support task-specific selection.

RQ2—platform cost. On the paired S/N configurations, the reComputer’s observed wall-clock training time and repeated CPU inference latency were higher than the AGX values. The median repeated mean-latency ratio was 4.40× (range 2.45–7.99×), while the median P95 ratio was 4.59× (range 2.51–7.76×). Training-time ratios remain operational outcomes under early stopping and should not be conflated with the repeated inference distributions.

RQ3—deployment operating points. Recalculation with repeated mean latency supports policy selection rather than a single winner: N-scale models anchor low-latency points, whereas task-dependent S/M models can improve strict-IoU accuracy where compute permits. AGX is the observed continuous/high-throughput tier; reComputer is the observed CPU baseline for lower-rate or triggered screening.

RQ4—Hailo deployment constraint. The validated reComputer measurements define the PyTorch CPU reference point. HailoRT inference could not be measured under the available study constraints because custom .pt checkpoints require .hef compilation through a supported Linux x86_64 build environment, whereas the local reComputer runtime is ARM64 and no compatible build host or managed conversion service was available. The correct interpretation is therefore CPU baseline characterization rather than Hailo-8 accelerator evaluation.

RQ5—event-candidate payload interface. The deterministic offline CPU replay demonstrated that checkpoint-derived detections can be transformed into schema-conformant ECEI event-candidate records. Across 5400 model-frame pairs, event JSON was 98.195% smaller than raw JPEG data by aggregate byte ratio, with task aggregates from 89.77% to 99.29%. Listings 1 and 2 provide complete paired detector-output and event-candidate examples. The result validates schema and byte reduction, not on-device serialization performance or network transport.

### 6.2. Implications for the ECEI Architecture

The benchmark supports a two-tier visual-compute policy within the proposed ECEI architecture. A reComputer node can screen selected frames and emit candidate detections, while an AGX node can process larger models, multiple streams, or escalated cases. This division is consistent with edge-computing principles [3,4,11]. The complete-pipeline extension will quantify event accuracy, communication savings, and observation-to-response latency alongside the detector evidence.

The Hailo-8 limitation is operationally important because accelerator deployability depends not only on the edge device but also on the availability of a compatible model-conversion and compilation environment. The present results therefore answer a practical deployment question: under a reComputer environment without x86_64 Hailo Dataflow Compiler access, custom YOLO models remain PyTorch CPU workloads. This finding should be presented as a deployment constraint and CPU reference condition rather than a negative accelerator benchmark.

### 6.3. Study Scope and Extension Pathways

The visual-compute benchmark now includes repeated on-device latency validation and an offline event-candidate payload replay. Extension priorities are (i) transport-aware measurement including protocol headers and retransmission, (ii) independent retraining replications to quantify checkpoint-to-checkpoint variability, (iii) power-normalized logging, and (iv) HailoRT characterization after a supported Linux x86_64 compilation path becomes available. Field studies should extend the analysis to adverse weather, full-video throughput, event-level false alarms, operator response, and rescue outcomes.

The fixed 320 × 320 protocol and fixed dataset partitions control input complexity across platforms. Subsequent field studies will extend the analysis to adverse weather, camera domains, energy, temperature, full-video throughput, network traffic, event-level false alarms, operator response, and rescue outcomes. A size-, occlusion-, and weather-stratified error study is also prioritized for Human Detection.

### 6.4. Threats to Validity

Replication validity: The 20 timing repetitions per image estimate runtime variability but not training uncertainty. The image subset was selected by deterministic SHA-256 ranking; its selection token is not interpreted as a random seed. Accuracy still reflects one archived checkpoint per configuration, so checkpoint-to-checkpoint or training-seed variance remains unquantified. The 17 byte-identical cross-platform checkpoint pairs and 98.18% unique-weight payload sensitivity result support replay consistency without implying independent model replications.

Hardware-path validity: Repeated AGX latency reflects synchronized CUDA-backed prediction on the Jetson device, and repeated reComputer latency reflects PyTorch CPU execution on aarch64. The latency comparison is deployment-realistic but not hardware-isolated. Because the two platforms require different native software stacks, including different PyTorch builds and execution backends, the reported reComputer/AGX latency ratios reflect the combined effect of hardware, operating system, framework build, backend availability, and runtime behavior. A hardware-isolated comparison would require additional ablation experiments with matched framework versions where possible, backend-specific profiling, and per-operator timing. The subsequent detector-to-event JSON replay was evaluated offline and is used to assess schema conformance and serialized byte size, not on-device event-serialization latency, edge-device runtime, or network transport performance.

Construct validity: Repeated detector latency covers prediction on pre-decoded images and is not camera-to-alert latency. It excludes acquisition, image decode, queueing, transport, retransmission, and central ingestion. The 98.195% event/raw reduction compares serialized JSON and JPEG byte lengths under an empty-envelope policy; it is neither serialization-time measurement nor full network-bandwidth reduction.

External validity: The 320 × 320 input size defines a compact edge-screening condition and may understate maximum attainable accuracy for small or partially occluded maritime targets. Public and provider-controlled datasets may not fully represent field cameras, weather, lighting, and vessel-domain variation.

## 7. Conclusions

This article benchmarks the visual-detection tiers of an Event-Centric Edge Intelligence architecture for maritime disaster response. Across 54 checkpoint configurations and 108,000 repeated predictions, AGX Orin supported the lowest complete-call latency range (19.84–57.09 ms mean), while reComputer established a 68.89–172.11 ms PyTorch CPU range for the compact tier. The CPU baseline anchors a future HailoRT campaign requiring compatible x86_64 compilation and on-device accelerator characterization.

Detector selection remains task- and tier-dependent: YOLOv8-M led Human and Ship Detection on AGX, YOLO26-M led Marine Trash Detection on AGX, and YOLOv8-S led all three reComputer tasks. Repeated S/N latency ratios had a 4.40× median for means and 4.59× for P95 values. A separate offline CPU replay converted all 54 logical checkpoint outputs into verified ECEI JSON records and measured 98.20% aggregate event/raw byte reduction. These results validate the detector operating tiers and detector-to-event schema, not camera-to-alert latency, on-device serialization speed, Hailo-8 acceleration, or a complete maritime response system.

## Figures and Tables

**Table 1:** Verification status of the mathematical expressions used in this article.

Equation(s)	Definition	Status	Evidence in This Study
(1)	Temporal observation selection	Conceptual	Reserved for synchronized multimodal validation
(2)	Reliability-weighted encoding	Conceptual	Reserved for reliability-model validation
(3)	Fused representation and event probabilities	Conceptual	Reserved for multimodal fusion validation
(4)	Visual detections to event candidates	Interface protocol measured	Offline CPU replay produced and verified 5400 detector JSON and 5400 event-candidate JSON messages from 300 deterministically selected validation frames across 54 logical checkpoint slots
(5)	Operational priority score	Conceptual	Reserved for outcome-calibrated priority validation
(6)	Union of node-level event sets	Conceptual	Reserved for end-to-end node integration
(7)	Communication reduction and end-to-end latency	Payload and detector processing measured	Event/raw byte reduction was 98.20% overall; repeated detector processing was measured on both edge devices, while camera-to-central network latency remains unmeasured
(8)–(11)	Detection metrics and Pareto rule	Empirical	Accuracy from 54 archived checkpoints; Pareto analysis recalculated using 108,000 repeated latency observations

**Table 2:** Hardware platforms and exact measured execution paths.

Platform	Compute and Memory	Model Coverage	Measured Path	Interpretation
Jetson AGX Orin 64 GB Developer Kit(NVIDIA Corporation, Santa Clara, CA, USA)	12-core Arm CPU; 2048-core Ampere GPU; 64 GB unified memory	YOLO26/11/v8: L, M, S, N	GPU-backed training; synchronized repeated CUDA inference	High-throughput edge server
reComputer AI R2000-12(Seeed Studio, Shenzhen, China)	Raspberry Pi 5; 16 GB; Hailo-8 inference accelerator	YOLO26/11/v8: S, N	PyTorch CPU training and repeated inference on aarch64	CPU baseline; HailoRT not measured

**Table 3:** Dataset composition used in the benchmark.

Task	Images	Train	Validation	Test	Classes	Split	Preprocessing
Human Detection	6231	5448	522	261	1	87.4/8.4/ 4.2	Auto-orient, resize, exposure and noise augmentation
Ship Detection	6941	4861	1389	691	6	70/20/10	Auto-orient and resize
Marine Trash Detection	1400	979	280	141	11	70/20/10	Resize to benchmark input

**Table 4:** Training controls and repeated inference-validation design.

Parameter	Setting	Control Level	Design Role
Epoch cap	100	Yes	Upper bound paired with patience-based stopping
Patience	5 epochs	Yes	Common early-stopping criterion
Batch/input	2/320 × 320	Yes	Common computational input across platforms
Inference replication	100 images × 20 measured repetitions	Yes	Distributional runtime characterization; warm-up excluded
Workers/AMP	0/True	Yes	Common loading and precision policy
Optimizer	auto → AdamW	Policy fixed	Common framework optimizer policy
Initial learning rate	Human Detection 0.002; Ship Detection 0.001; Marine Trash Detection 0.000667	Task specific	Resolved by the common optimizer policy
Momentum	0.9	Policy resolved	Resolved by the common optimizer policy
Model coverage	AGX L/M/S/N; reComputer S/N	Tier specific	Deployment-oriented platform coverage

**Table 5:** Software environments used in the benchmark.

Platform	Framework	Python/PyTorch	Measured Execution Environment
Jetson AGX Orin	Ultralytics 8.4.31	3.8.10/2.1.0a0+41361538.nv23.06	CUDA 11.4; GPU-backed training and validation
reComputer training	Ultralytics 8.4.35	3.11.2/2.11.0+cpu	Linux aarch64; PyTorch CPU

**Table 6:** Accuracy–latency Pareto candidates recalculated using repeated mean complete-call latency.

Task	AGX Pareto Candidates	reComputer Pareto Candidates	Repeated Validation
Human Detection	YOLOv8-N; YOLO26-S/M; YOLOv8-M	YOLOv8-N/S	2000 observations per checkpoint
Ship Detection	YOLOv8-N/S; YOLO26-M; YOLO11-M; YOLOv8-M	YOLOv8-N; YOLO26-N; YOLOv8-S	2000 observations per checkpoint
Marine Trash Detection	YOLOv8-N/S/M; YOLO26-S/M	YOLOv8-N/S; YOLO11-N; YOLO26-N	2000 observations per checkpoint

**Table 7:** Highest observed $mAP_{50–95}$ configuration for each task and device with repeated latency statistics.

Task	Device	Model	P	R	$mAP_{50}$	$mAP_{50–95}$	Repeated Mean/P95 (ms)
Human Detection	AGX Orin	YOLOv8-M	0.750	0.692	0.744	0.331	32.9/33.6
Human Detection	reComputer	YOLOv8-S	0.744	0.674	0.724	0.318	160.3/161.9
Ship Detection	AGX Orin	YOLOv8-M	0.957	0.965	0.985	0.813	32.7/33.6
Ship Detection	reComputer	YOLOv8-S	0.957	0.961	0.986	0.806	159.7/161.5
Marine Trash Detection	AGX Orin	YOLO26-M	0.764	0.737	0.787	0.639	32.6/33.9
Marine Trash Detection	reComputer	YOLOv8-S	0.724	0.652	0.708	0.558	141.0/162.1

**Table 8:** Repeated reComputer-to-AGX latency ratios on the paired S/N subset.

Scope	Paired Configurations	Median Mean Ratio	Mean-Ratio Range	Median P95 Ratio	P95-Ratio Range
Human Detection	6	4.97×	2.81–7.94×	4.81×	2.51–7.76×
Ship Detection	6	4.97×	3.09–7.99×	4.82×	3.01–7.76×
Marine Trash Detection	6	4.06×	2.45–6.34×	4.46×	2.78–6.79×
All tasks	18	4.40×	2.45–7.99×	4.59×	2.51–7.76×

**Table 9:** Measured payload sizes and event-candidate/raw-frame reduction by task and checkpoint-origin group for the complete 54-configuration offline CPU replay. Byte values are means over model-frame pairs; every model for a task used the same 100 JPEGs.

Task	Checkpoint Origin	Logical Models	Model-Frame Pairs	Raw JPEG Mean (Bytes)	Detector JSON Mean (Bytes)	Event JSON Mean (Bytes)	Event/Raw Reduction (%)
Human Detection	AGX Orin	12	1200	56,400.3	1367.8	6211.7	88.986
Human Detection	reComputer	6	600	56,400.3	1137.0	4892.4	91.326
Ship Detection	AGX Orin	12	1200	45,510.0	392.8	762.1	98.325
Ship Detection	reComputer	6	600	45,510.0	391.1	753.1	98.345
Marine Trash	AGX Orin	12	1200	426,985.7	834.4	3282.1	99.231
Marine Trash	reComputer	6	600	426,985.7	674.1	2479.8	99.419
All tasks	AGX Orin	36	3600	176,298.7	865.0	3418.6	98.061
All tasks	reComputer	18	1800	176,298.7	734.1	2708.4	98.464
All tasks	All devices	54	5400	176,298.7	821.4	3181.9	98.195

**Table 10:** Model-by-model payload comparison on the paired S/N subset. Accepted detections are totals over the same 100 task-specific JPEGs; Event JSON values are per-frame means.

Task	Model	Accepted Detections		Event JSON Mean (Bytes)		Event/Raw Reduction (%)	
		AGX Origin	reComputer Origin	AGX Origin	reComputer Origin	AGX Origin	reComputer Origin
Human Detection	YOLO26-S	979	979	6150.0	6150.0	89.096	89.096
Human Detection	YOLO26-N	476	476	3077.7	3077.7	94.543	94.543
Human Detection	YOLO11-S	682	682	4334.9	4334.9	92.314	92.314
Human Detection	YOLO11-N	778	778	4922.0	4922.0	91.273	91.273
Human Detection	YOLOv8-S	889	889	5599.8	5599.8	90.071	90.071
Human Detection	YOLOv8-N	835	835	5270.0	5270.0	90.656	90.656
Ship Detection	YOLO26-S	97	97	735.3	735.3	98.384	98.384
Ship Detection	YOLO26-N	87	87	675.3	675.3	98.516	98.516
Ship Detection	YOLO11-S	100	100	752.9	752.9	98.346	98.346
Ship Detection	YOLO11-N	107	107	795.0	795.0	98.253	98.253
Ship Detection	YOLOv8-S	108	108	801.2	801.2	98.240	98.240
Ship Detection	YOLOv8-N	101	101	759.0	759.0	98.332	98.332
Marine Trash	YOLO26-S	520	520	3022.5	3022.5	99.292	99.292
Marine Trash	YOLO26-N	425	425	2491.2	2491.2	99.417	99.417
Marine Trash	YOLO11-S	567	567	3286.2	3286.2	99.230	99.230
Marine Trash	YOLO11-N	504	504	2932.5	2932.5	99.313	99.313
Marine Trash	YOLOv8-S	532	532	3088.1	3088.1	99.277	99.277
Marine Trash	YOLOv8-N	505	505	2937.6	2937.6	99.312	99.312

## Data Availability

The Human Detection and Ship Detection source datasets are available from the versioned Roboflow Universe resources cited in [26,27] under the Creative Commons Attribution 4.0 International license (CC BY 4.0). The Marine Trash Detection data were obtained from the AI Hub dataset cited in [28] and organized into project-specific experimental partitions. AI Hub retains access control over the source images and annotations, and redistribution of the converted labels or organized derivative requires provider authorization. Researchers may apply directly through AI Hub. Aggregate experimental results and analysis code that do not contain restricted source data may be made available by the corresponding author upon reasonable request. Public release of trained model weights is governed by source-data and deployment restrictions.

## Appendix A. Complete Empirical Results

Table A1, Table A2 and Table A3 report training and detector-accuracy results for every configuration exactly once. Their final inference column is retained only as a legacy provenance field from the archived training summaries and is not used for the latency conclusions. Table A4, Table A5 and Table A6 supersede that field with the complete repeated on-device latency distributions. “Selected epoch” is the epoch with maximum validation $mAP_{50–95}$ for AGX or the best epoch reported during reComputer training.

**Table A1:** Complete benchmark results for Human Detection.

Device	Model	Epoch (Best/Done)	Train (h)	P	R	$mAP_{50}$	$mAP_{50–95}$	Legacy Val. Inf. (ms)
AGX Orin	YOLO26-L	89/94	25.275	0.735	0.660	0.729	0.330	24.4
AGX Orin	YOLO26-M	90/95	20.962	0.736	0.665	0.732	0.327	19.1
AGX Orin	YOLO26-S	99/100	20.716	0.726	0.638	0.709	0.313	12.2
AGX Orin	YOLO26-N	45/50	10.416	0.643	0.578	0.623	0.255	8.4
AGX Orin	YOLO11-L	87/92	31.258	0.727	0.655	0.703	0.309	32.1
AGX Orin	YOLO11-M	79/84	14.450	0.726	0.661	0.713	0.315	19.0
AGX Orin	YOLO11-S	23/28	4.140	0.676	0.610	0.639	0.264	10.7
AGX Orin	YOLO11-N	79/84	11.999	0.696	0.619	0.673	0.285	5.9
AGX Orin	YOLOv8-L	87/92	18.642	0.745	0.708	0.746	0.331	26.0
AGX Orin	YOLOv8-M	87/92	14.018	0.750	0.692	0.744	0.331	17.3
AGX Orin	YOLOv8-S	37/42	5.094	0.702	0.633	0.673	0.286	9.4
AGX Orin	YOLOv8-N	84/89	10.921	0.709	0.626	0.679	0.291	4.8
reComputer	YOLO26-S	100/100	114.893	0.722	0.648	0.709	0.309	175.4
reComputer	YOLO26-N	100/100	54.746	0.662	0.593	0.650	0.276	77.5
reComputer	YOLO11-S	100/100	108.547	0.734	0.647	0.707	0.311	180.8
reComputer	YOLO11-N	55/60	29.840	0.690	0.615	0.657	0.274	78.7
reComputer	YOLOv8-S	93/98	120.746	0.744	0.674	0.724	0.318	175.0
reComputer	YOLOv8-N	80/85	43.704	0.708	0.625	0.675	0.288	72.5

**Table A2:** Complete benchmark results for Ship Detection.

Device	Model	Epoch (Best/Done)	Train (h)	P	R	$mAP_{50}$	$mAP_{50–95}$	Legacy Val. Inf. (ms)
AGX Orin	YOLO26-L	26/31	7.638	0.921	0.882	0.952	0.704	24.6
AGX Orin	YOLO26-M	71/76	15.535	0.948	0.942	0.981	0.792	19.1
AGX Orin	YOLO26-S	61/66	12.524	0.945	0.933	0.975	0.764	11.6
AGX Orin	YOLO26-N	44/49	9.224	0.889	0.866	0.941	0.706	7.1
AGX Orin	YOLO11-L	60/65	13.591	0.955	0.915	0.976	0.767	24.0
AGX Orin	YOLO11-M	89/94	15.090	0.968	0.961	0.984	0.796	18.8
AGX Orin	YOLO11-S	45/50	6.848	0.920	0.930	0.970	0.740	10.5
AGX Orin	YOLO11-N	84/89	11.956	0.955	0.917	0.973	0.757	5.8
AGX Orin	YOLOv8-L	43/48	9.124	0.944	0.933	0.976	0.753	25.9
AGX Orin	YOLOv8-M	88/93	13.368	0.957	0.965	0.985	0.813	17.0
AGX Orin	YOLOv8-S	68/73	8.352	0.956	0.941	0.979	0.781	9.3
AGX Orin	YOLOv8-N	67/72	8.275	0.946	0.912	0.970	0.750	4.3
reComputer	YOLO26-S	89/94	103.277	0.954	0.938	0.983	0.785	182.4
reComputer	YOLO26-N	50/55	27.487	0.916	0.877	0.953	0.727	77.1
reComputer	YOLO11-S	43/48	48.549	0.928	0.928	0.970	0.730	180.3
reComputer	YOLO11-N	40/45	20.716	0.936	0.889	0.950	0.697	79.6
reComputer	YOLOv8-S	100/100	114.549	0.957	0.961	0.986	0.806	174.9
reComputer	YOLOv8-N	42/47	22.272	0.936	0.892	0.957	0.708	72.5

**Table A3:** Complete benchmark results for Marine Trash Detection.

Device	Model	Epoch (Best/Done)	Train (h)	P	R	$mAP_{50}$	$mAP_{50–95}$	Legacy Val. Inf. (ms)
AGX Orin	YOLO26-L	46/51	3.189	0.781	0.716	0.781	0.628	19.5
AGX Orin	YOLO26-M	67/72	3.844	0.764	0.737	0.787	0.639	15.4
AGX Orin	YOLO26-S	33/38	1.893	0.723	0.646	0.685	0.527	9.3
AGX Orin	YOLO26-N	40/45	2.197	0.614	0.547	0.575	0.430	7.4
AGX Orin	YOLO11-L	39/44	2.337	0.746	0.647	0.706	0.561	19.3
AGX Orin	YOLO11-M	51/56	2.443	0.738	0.684	0.734	0.584	15.1
AGX Orin	YOLO11-S	32/37	1.429	0.689	0.625	0.676	0.519	8.4
AGX Orin	YOLO11-N	23/28	1.113	0.562	0.546	0.552	0.405	6.1
AGX Orin	YOLOv8-L	31/36	1.783	0.692	0.644	0.695	0.537	21.1
AGX Orin	YOLOv8-M	40/45	1.793	0.761	0.652	0.714	0.568	14.0
AGX Orin	YOLOv8-S	32/37	1.242	0.683	0.651	0.676	0.522	7.5
AGX Orin	YOLOv8-N	23/28	0.970	0.556	0.572	0.558	0.413	4.7
reComputer	YOLO26-S	11/16	3.667	0.599	0.565	0.589	0.440	152.9
reComputer	YOLO26-N	69/74	8.166	0.672	0.573	0.622	0.474	65.4
reComputer	YOLO11-S	28/33	7.014	0.672	0.622	0.659	0.498	149.6
reComputer	YOLO11-N	40/45	4.572	0.600	0.584	0.592	0.442	66.3
reComputer	YOLOv8-S	51/56	13.346	0.724	0.652	0.708	0.558	145.3
reComputer	YOLOv8-N	32/37	3.858	0.641	0.547	0.586	0.434	60.7

### Complete Repeated On-Device Latency Results

Table A4, Table A5 and Table A6 report the complete repeated on-device latency distributions for the three detection tasks.

**Table A4:** Repeated on-device latency distribution for Human Detection (100 images × 20 repetitions per checkpoint).

Device	Model	n	Images × Reps	Mean (ms)	P50 (ms)	P95 (ms)	SD (ms)	FPS
AGX Orin	YOLO26-L	2000	100 × 20	42.20	42.11	43.01	0.42	23.70
AGX Orin	YOLO26-M	2000	100 × 20	30.58	30.41	31.49	0.52	32.70
AGX Orin	YOLO26-S	2000	100 × 20	28.15	28.08	29.11	0.48	35.52
AGX Orin	YOLO26-N	2000	100 × 20	27.41	27.29	28.38	0.45	36.49
AGX Orin	YOLO11-L	2000	100 × 20	57.09	57.34	61.46	3.00	17.52
AGX Orin	YOLO11-M	2000	100 × 20	43.57	43.67	48.94	3.25	22.95
AGX Orin	YOLO11-S	2000	100 × 20	25.23	25.09	26.60	0.70	39.64
AGX Orin	YOLO11-N	2000	100 × 20	27.97	27.64	32.18	2.74	35.76
AGX Orin	YOLOv8-L	2000	100 × 20	47.60	47.48	48.46	0.65	21.01
AGX Orin	YOLOv8-M	2000	100 × 20	32.87	32.79	33.62	0.44	30.42
AGX Orin	YOLOv8-S	2000	100 × 20	20.19	20.12	20.87	0.42	49.53
AGX Orin	YOLOv8-N	2000	100 × 20	20.00	19.84	21.33	0.56	50.01
reComputer	YOLO26-S	2000	100 × 20	172.11	171.90	174.72	2.61	5.81
reComputer	YOLO26-N	2000	100 × 20	84.14	84.00	84.86	1.46	11.88
reComputer	YOLO11-S	2000	100 × 20	163.01	162.79	166.18	3.29	6.13
reComputer	YOLO11-N	2000	100 × 20	78.66	78.17	80.91	1.93	12.71
reComputer	YOLOv8-S	2000	100 × 20	160.30	160.04	161.93	2.15	6.24
reComputer	YOLOv8-N	2000	100 × 20	76.52	76.40	77.15	1.45	13.07

**Table A5:** Repeated on-device latency distribution for Ship Detection (100 images × 20 repetitions per checkpoint).

Device	Model	n	Images × Reps	Mean (ms)	P50 (ms)	P95 (ms)	SD (ms)	FPS
AGX Orin	YOLO26-L	2000	100 × 20	42.49	42.38	43.35	0.46	23.54
AGX Orin	YOLO26-M	2000	100 × 20	30.51	30.40	31.36	0.42	32.78
AGX Orin	YOLO26-S	2000	100 × 20	28.15	28.11	29.17	0.48	35.53
AGX Orin	YOLO26-N	2000	100 × 20	27.50	27.38	28.48	0.49	36.36
AGX Orin	YOLO11-L	2000	100 × 20	43.73	43.65	44.68	0.53	22.87
AGX Orin	YOLO11-M	2000	100 × 20	30.61	30.54	31.95	0.76	32.67
AGX Orin	YOLO11-S	2000	100 × 20	25.32	25.21	26.78	0.77	39.50
AGX Orin	YOLO11-N	2000	100 × 20	25.10	25.06	26.34	0.73	39.83
AGX Orin	YOLOv8-L	2000	100 × 20	47.50	47.52	48.60	0.71	21.05
AGX Orin	YOLOv8-M	2000	100 × 20	32.73	32.80	33.65	0.66	30.55
AGX Orin	YOLOv8-S	2000	100 × 20	20.00	20.05	20.82	0.63	50.00
AGX Orin	YOLOv8-N	2000	100 × 20	19.84	19.80	21.13	0.72	50.39
reComputer	YOLO26-S	2000	100 × 20	172.06	171.83	174.84	2.54	5.81
reComputer	YOLO26-N	2000	100 × 20	84.88	84.64	85.83	1.42	11.78
reComputer	YOLO11-S	2000	100 × 20	164.50	164.12	166.88	2.46	6.08
reComputer	YOLO11-N	2000	100 × 20	81.44	81.57	83.08	1.86	12.28
reComputer	YOLOv8-S	2000	100 × 20	159.70	159.39	161.48	2.23	6.26
reComputer	YOLOv8-N	2000	100 × 20	76.12	75.97	76.95	1.41	13.14

**Table A6:** Repeated on-device latency distribution for Marine Trash Detection (100 images × 20 repetitions per checkpoint).

Device	Model	n	Images × Reps	Mean (ms)	P50 (ms)	P95 (ms)	SD (ms)	FPS
AGX Orin	YOLO26-L	2000	100 × 20	44.32	44.45	45.81	1.18	22.56
AGX Orin	YOLO26-M	2000	100 × 20	32.57	32.72	33.88	0.89	30.70
AGX Orin	YOLO26-S	2000	100 × 20	30.06	30.05	31.46	1.50	33.26
AGX Orin	YOLO26-N	2000	100 × 20	29.48	29.60	30.98	1.93	33.92
AGX Orin	YOLO11-L	2000	100 × 20	45.95	46.03	47.22	5.81	21.76
AGX Orin	YOLO11-M	2000	100 × 20	32.84	32.96	34.34	1.19	30.45
AGX Orin	YOLO11-S	2000	100 × 20	27.47	27.50	28.73	3.05	36.41
AGX Orin	YOLO11-N	2000	100 × 20	27.14	27.17	28.33	4.45	36.85
AGX Orin	YOLOv8-L	2000	100 × 20	46.20	44.95	50.53	3.69	21.64
AGX Orin	YOLOv8-M	2000	100 × 20	32.55	31.35	36.16	5.19	30.72
AGX Orin	YOLOv8-S	2000	100 × 20	22.26	22.30	23.89	1.41	44.92
AGX Orin	YOLOv8-N	2000	100 × 20	21.85	21.85	23.28	1.18	45.78
reComputer	YOLO26-S	2000	100 × 20	149.36	131.36	174.03	21.27	6.70
reComputer	YOLO26-N	2000	100 × 20	72.16	63.09	86.19	11.33	13.86
reComputer	YOLO11-S	2000	100 × 20	143.28	125.08	167.43	21.36	6.98
reComputer	YOLO11-N	2000	100 × 20	71.56	62.98	83.93	10.71	13.97
reComputer	YOLOv8-S	2000	100 × 20	141.03	124.54	162.14	18.99	7.09
reComputer	YOLOv8-N	2000	100 × 20	68.89	61.48	78.80	8.85	14.52
